# The Regulatory Role of GBF1 on Osteoclast Activation Through EIF2a Mediated ER Stress and Novel Marker FAM129A Induction

**DOI:** 10.3389/fcell.2021.706768

**Published:** 2021-08-25

**Authors:** Cailing Wen, Yuheng Zhou, Yanting Xu, Huijing Tan, Caixia Pang, Haiqian Liu, Kaifei Liu, Linlin Wei, Hui Luo, Tian Qin, Chonghua He, Cuiling Liu, Chun Zhou

**Affiliations:** ^1^SMU-KI United Medical Inflammatory Center, Guangdong Provincial Key Laboratory of Shock and Microcirculation, School of Pharmaceutical Sciences, Southern Medical University, Guangzhou, China; ^2^School of Medicine, Sun Yat-sen University, Shenzhen, China; ^3^Department of Pharmacy, Jingzhou Central Hospital, Jingzhou, China; ^4^Shenzhen Bao’an District Traditional Chinese Medicine Hospital, Shenzhen, China

**Keywords:** osteoclast, GBF1, bone, endoplasmic reticulum stress, autophagy

## Abstract

Bone-resorbing activities of osteoclasts (OCs) are highly dependent on actin cytoskeleton remodeling, plasma membrane reorganization, and vesicle trafficking pathways, which are partially regulated by ARF-GTPases. In the present study, the functional roles of Golgi brefeldin A resistance factor 1 (GBF1) are proposed. GBF1 is responsible for the activation of the ARFs family and vesicular transport at the endoplasmic reticulum–Golgi interface in different stages of OCs differentiation. In the early stage, GBF1 deficiency impaired OCs differentiation and was accompanied with OCs swelling and reduced formation of mature OCs, indicating that GBF1 participates in osteoclastogenesis. Using siRNA and the specific inhibitor GCA for GBF1 knockdown upregulated endoplasmic reticulum stress-associated signaling molecules, including BiP, p-PERK, p-EIF2α, and FAM129A, and promoted autophagic Beclin1, Atg7, p62, and LC3 axis, leading to apoptosis of OCs. The present data suggest that, by blocking COPI-mediated vesicular trafficking, GBF1 inhibition caused intense stress to the endoplasmic reticulum and excessive autophagy, eventually resulting in the apoptosis of mature OCs and impaired bone resorption function.

## Introduction

Bone homeostasis is finely regulated through spatial and temporal control of the bone remodeling process, which is a repetitive cycle of osteoblastic bone formation and osteoclastic bone resorption (Kim et al., 2020). Imbalance of remodeling leads to disruption of bone mass and structure and can potentially result in numerous bone metabolic diseases, such as osteoporosis (Pekkinen et al., 2019), rheumatoid arthritis (RA) (Tateiwa et al., 2019), osteosclerosis (Zhu et al., 2016), and bone-related tumors (Jimenez-Andrade et al., 2010). Osteoclasts (OCs) are multinucleated giant cells developed from multiple monocytes under the stimulation of macrophage colony-stimulating factor (M-CSF) and receptor activator of NF-κB ligand (RANKL) (Teitelbaum and Ross, 2003). RANKL stimulation activates components of the NF-κB and MAPK signaling pathways and various transcription factors, including c-Fos and T cell nuclear factor 1 (NFATc1) (Takayanagi et al., 2002; Ihn et al., 2015). Through RANKL, c-Fos regulates the induction of NFATc1 expression, and the cooperation between c-Fos and NFATc1 induces the expression of downstream factors including tartrate-resistant acid phosphatase (TRAP), cathepsin K (CtsK), matrix metalloproteinase (MMP-2/9), and α_v_β_3_-Integrin, and eventually damages the bone structure (Quan et al., 2015).

As a guanine-nucleotide exchange factor (GEFs) containing a conserved catalytic Sec7 domain, GBF1 activates the ADP-ribosylation factors (ARFs) family of small GTPases. Through replacing GDP with GTP for subsequent recruitment of different vesicle coating proteins in different compartments, the conversion of activated ARFs is facilitated by GBF1 being preferentially concentrated at vesicular and tubular structures apposed to the *cis*-Golgi network, endoplasmic reticulum–Golgi intermediate compartment (ERGIC), and minor fractions to the Golgi stacks (Zhao et al., 2006). To regulate the recruitment of coatomer protein complex (COPI) onto the membranes, class I (ARF1 and ARF3), especially ARF1, is controlled by GBF1 localized within *cis*-Golgi and Golgi apparatus (Golinelli-Cohen et al., 2009), whereas GBF1 on the *trans*-Golgi network (TGN) and endosomes recruit the AP-1 clathrin adaptor complex and the AP-3 complex, respectively (Lowery et al., 2013). Thus, in addition to directly acting on vesicular transport in the early secretory pathway, GBF1 also has a significant impact on maintaining the structural integrity and dynamic alteration of the Golgi apparatus (Magliozzi et al., 2018). Furthermore, emerging evidence has revealed that GBF1 regulated different cycles of RNA replication in several virus families (Martínez and Arias, 2020), participated in mitochondrial migration and localization (Ackema et al., 2014), and affected the occurrence and development of neurodegenerative diseases (Mendoza-Ferreira et al., 2020).

The unfolded protein response (UPR) is a protective mechanism for endoplasmic reticulum (ER) responding to internal and external stimuli, enhancing the folding ability of proteins, suspending the translation of most proteins, and accelerating degradation. However, the stress to endoplasmic reticulum (ERS) is ultimately triggered by persistent or intense UPR (Hetz et al., 2020). Presently, the PRKR-like ER kinase (PERK)–eukaryotic translation initiation factor 2α (EIF2α) axis is regarded as the central signaling pathway of UPR. Misfolded protein in the ER lumen binds with BiP and is released from the UPR sensors, thereby further promoting PERK oligomerization and self-phosphorylation. The process activates EIF2α and widely downregulates the mRNA transcription and protein synthesis. Additionally, p-EIF2α paradoxically upregulates the expression of ATF4 in order to increase the capacity for protein transport, antioxidant response, amino acid synthesis, and autophagy following nuclear translocation (Horiuchi et al., 2016). In prostate cancer cells, Family With Sequence Similarity 129 Member A (FAM129A) activated upon ER stress is a novel direct ATF4-C/EBPβ target gene (Pällmann et al., 2019). FAM129A consistently upregulates the pro-apoptotic protein Bax, decreases anti-apoptotic protein Bcl-2 level by activating CCAAT/enhancer-binding protein (C/EBP) homologous protein (CHOP), and finally leads to the release of mitochondrial cytochrome c, the formation of apoptotic bodies, and the cleavage of caspase-3 to induce apoptosis (Ding et al., 2018). At the same time, FAM129A also regulates the PERK–EIF2α pathway that allows the cell to fine-tune the response thereof to afferent pressure depending on the environment through both positive and negative feedback loops (Evstafieva et al., 2018).

Serving as one of the more recent effector mechanisms for UPR, autophagy is an intracellular degradation mechanism that delivers cytoplasmic constituents to lysosomes and has an extensive variety of physiological and pathophysiological functions (Filomeni et al., 2015). Baseline autophagy protects cells from injury and contributes to cell survival, while rapid or severe autophagy results in programmed cell death (Li et al., 2019; Song et al., 2019). Autophagy has recently been demonstrated to be involved in OC differentiation, bone destruction, and inflammation depending on the evolution of the autophagosome structure (Tong et al., 2018). Autophagy-related proteins, such as Beclin1, Atg5, p62, and LC3, are significantly responsible for the autophagy nucleation process, autophagosome elongation, and maturation stage (Montaseri et al., 2020).

As evidenced by recent studies, ERS was linked to the progression of skeletal disorders. In previous research by the present authors, inhibition of GBF1 was shown to induce ERS in SH-SY5Y cells (Liu et al., 2019), which is consistent with the upregulation of UPR and activated ERS response element gene caused by deletion of GBF1 in some creatures (Kim et al., 2018). Therefore, GBF1 was proposed to be the pivotal ER sensor to monitor ERS in OCs through the PERK–EIF2α axis, control activation of OC-related transcription factors, and affect the normal differentiation of OCs.

## Materials and Methods

### Reagents and Antibodies

RAW264.7 cell line was obtained from ATCC (Manassas, VA, United States). Control and GBF1 siRNA (E11) were designed and synthesized by GenePharma (Suzhou, China). Dulbecco’s modified Eagle’s medium, fetal bovine serum (FBS), Opti-MEM^®^ I Reduced-Serum Medium, penicillin, and streptomycin were obtained from Gibco (Rockville, MD, United States). Soluble recombinant mouse RANKL, Golgicide A (GCA), and TRAP staining kit were purchased separately from PeproTech (Rocky Hill, NJ, United States), Selleck (Shanghai, China), and Sigma-Aldrich (St. Louis, MO, United States). Antibodies against NFATc1, V-ATPase, EIF2α, p-EIF2α, PERK, p-PERK, Atg5, LC3A/B, GAPDH, Cleaved Caspase-3, Caspase-3, Bax, Bcl-xL, Cytochrome C, and LAMP1 were obtained from Cell signaling Technology. Antibodies against c-Fos, MMP9, Beclin1, and p62 were obtained from Abcam. Antibodies against GBF1, BiP, and EEA1 were obtained from BD Transduction Laboratories^TM^. Antibodies against FAM129A, Rab7, Bcl-2, and β-tubulin were obtained from Proteintech.

### Cell Culture and GCA Treatment

RAW264.7 cells were cultured in DMEM containing 10% FBS at 37°C with 5% CO_2_. For the formal experiments, cells were cultured in the presence of RANKL (50 ng/ml) with or without different concentrations of GCA (0, 1.5, 2, and 2.5 μM) in a six-well plate (3 × 10^4^ cells/well) for 56 days, with the medium being changed every other day.

### siRNA Knockdown

According to the manufacturer’s instructions, the siRNA sequence targeting GBF1 (NM_178930.3) or the negative control was transfected into RAW264.7 cells using Lipofectamine^TM^ 2000 (Invitrogen). The gene ID number of GBF1 is 8729. Cells were collected at the logarithmic growth phase and the cell suspension was adjusted to 1 × 10^4^/ml, inoculated in a six-well plate, and placed in a 5% CO_2_ incubator at 37°C for 12 h. When the cell density reached 70–80% confluence, 5 μl of lipo2000 was added to 250 μl of Opti-MEM and mixed well, followed by the addition of 250 μl of Opti-MEM mixed with 100 pmol siRNA, which was incubated for a further 5 min at room temperature (RT). The lipo2000 was mixed with the siRNAs and incubated for 20 min at RT. The serum-containing medium in the six-well plate was removed, before 1.5 ml of serum-free medium containing siRNAs was added and cultured in a 5% CO_2_ incubator at 37°C. The solution was changed after 6 h to a normal medium (Xu et al., 2021).

### Cell Viability Assay

RAW264.7 cells were cultured in a 96-well plate (7 × 10^4^/ml) and treated with or without GCA (0, 0.5, 1, 1.5, 2, and 2.5 μM) for 72 h. Thereafter, CCK8 kit solution was added to each well. Absorbance was measured at 450 nm using an ELISA microplate reader after 3 h of incubation at 37°C in 5% CO_2_ (BioTek Synergy H1, MA, United States). Cell viability was presented as the percentage of control.

### Bone Resorption Assay

RAW264.7 cells were inoculated in the Osteo Assay Surface 96-well plate (Corning, St. Lowell, MA, United States) and OC differentiation was induced according to the aforementioned methods. Subsequently, the cells were washed with 10% bleach solution and air dried for 3–5 h at RT. The resorption pits were captured under a light microscope (Nikon, Tokyo, Japan). The bone absorption area of each group was calculated by Image-Pro-Plus 6 software.

### Tartrate-Resistant Acid Phosphatase Assay

The staining reagents were prepared according to the manufacturer’s instructions. RAW264.7 cells were fixed with 10% formalin at RT for 10 min and incubated in staining solution at 37°C for 1.5 h in the dark. After washing three times with deionized water, hematoxylin was added to redyeing nucleus for 2–3 min. Using a light microscope, positive multinucleated OCs (nuclei ≧ 3) were observed and counted (Nikon, Tokyo, Japan).

### Acridine Orange Staining

RAW264.7 cells were inoculated in a 96-well plate, and after GBF1 knockout, the cells were induced to OCs and treated with 10 μg/ml acridine orange. After incubating at 37°C for 15 min, the cells were washed with PBS to remove any remaining dye. The fluorescence value was measured with a fluorescence microplate reader using 485-nm excitation wavelength and 588-nm emission wavelength (BioTek, Winooski, VT, United States). The intensity of fluorescence was directly proportional to the level of intracellular acidification.

### ELISA Assay

RAW264.7 cells were inoculated in a 24-well plate according to the aforementioned transfection and induction methods. After 5–7 days of culture, the medium was collected and centrifuged at 4°C and 3000 rpm for 20 min. The level of MMP9 was determined using ELISA kit according to the manufacturer’s instructions (Invitrogen, Carlsbad, CA, United States). Cytokine-specific antibody pre-coated plates were blocked with a blocking buffer for 1 h at RT, followed by incubation with the supernatant at 1:2 dilution. For detection, biotin-conjugated anti-mouse MMP9 antibodies, extravidin-horseradish peroxidase (HRP) conjugate, and TMB solution were used. The optical density at 450 nm was measured. The concentrations of MMP9 were calculated using a standard curve prepared according to the manufacturer’s instructions.

### Immunofluorescence Staining

RAW264.7 cells were cultured on glass cover slips according to the aforementioned description. The cells were harvested and fixed with 4% paraformaldehyde, permeabilized in 0.5% Triton X-100, and blocked with 10% normal goat serum in PBS. Subsequently, the cells were incubated with appropriate diluted primary antibodies (GBF1, FAM129A, and GM130, 1:100 in primary antibody diluent) for 1 h at RT, followed by incubation with Rhodamine Phalloidin (Invitrogen, Carlsbad, CA, United States) and Alexa Fluor-labeled secondary antibodies (1:1000 in blocking buffer) for 1 h in the dark. After washing with PBS, prolong^®^ Gold anti-fade reagent with DAPI (Molecular Probes, Waltham, MA, United States) was employed to counterstain the nuclei and the cells were inspected using a confocal microscope (Nikon, Tokyo, Japan) (Zhou et al., 2016).

### Quantitative PCR

The total RNA from cells was isolated with TRIzol reagent (Invitrogen Carlsbad, CA, United States). The RNA samples (0.5 pg–1 μg) were reverse transcribed into cDNA using Bestar^TM^ qPCR RT Kit (DBI^®^ Bioscience, Germany Quality). Quantitative real-time PCR analysis was performed in triplicate with a QuantiNova^TM^ SYBR^®^ Green PCR kit (QIAGEN) and detected by a LightCycler^®^ 480 Real-Time PCR System (Roche and Swiss). The thermal cycling conditions were as follows: 2 min at 95°C, followed by 40 cycles at 95°C for 5 s, 60°C for 10 s, and then 1 cycle at 95°C for 5 s, 65°C for 1 min, and finally slowly cooled down to 40°C and kept for 1 s. All the PCR reactions were performed using specific primers following the mouse sequences (Supplementary Table 1). To calculate the relative expression of each target gene, the comparative 2^–ΔΔCt^ method was used. The mRNA levels were normalized to levels of the endogenous housekeeping gene β-actin.

### Western Blot Analysis

RAW264.7 cells were homogenized on ice in radioimmune precipitation assay (RIPA) buffer containing protease inhibitors and phosphatase inhibitors for 30 min. The lysates were centrifuged at 12,000 × *g* for 15 min at 4°C, and the supernatant was collected to determine the protein concentration using a Pierce^TM^ BCA protein assay kit (Thermo Scientific^TM^). Isolated proteins (10 μl and 30–35 μg) were fractioned using 10% SDS-PAGE and electro-transferred to polyvinylidene fluoride membranes (Bio-Rad, Hercules, CA, United States). After blocking with 5% skim milk dissolved in TBST buffer for 1 h at RT, membranes were incubated with primary antibodies (1:500 for MMP9, GM130; 1:1,000 for GBF1, c-Fos, NFATc1, V-ATPase, CtsK, COPGI, FAM129A, EIF2α/p-EIF2α, PERK/p-PERK, Beclin1, p62, Atg5, LC3A/B, GAPDH, Cleaved Caspase-3, Caspase-3, Bax, Bcl-2, Bcl-xL, Cytochrome C, EEA1, Rab7, and LAMP1; 1:5,000 for BiP, β-tubulin) overnight at 4°C and then probed with horseradish peroxidase-conjugated secondary anti-rabbit and anti-mouse antibodies for 1 h at RT. Signals were detected with enhanced chemiluminescence (ECL) and visualized using ChemiDoc^TM^ Touch Imaging System (Bio-Rad, Hercules, CA, United States). The band density was analyzed using the Image J software. Data were presented with reference to control intensities of GAPDH or β-tubulin.

### Statistical Analysis

All data were expressed as mean ± SD (*n* = 3, each with duplicates). The results were assessed with one-way ANOVA followed by Dunnett’s *post hoc* test, with differences of *p*-value < 0.05 being considered statistically significant.

## Results

### GBF1 Mediated OCs Activation and Bone Absorption

**FIGURE 1:**
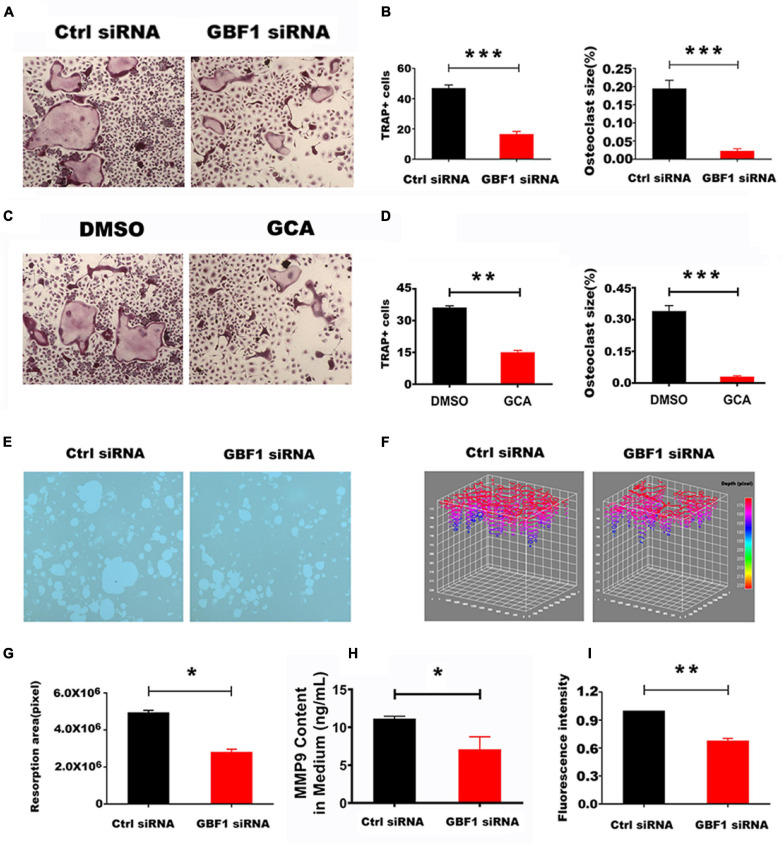
Effect of GBF1 on osteoclast differentiation and function *in vitro*. **(A,C)** RAW264.7 cells were transfected with scrambled siRNA (NC) as control, or with GBF1 siRNA E11. Cells were cultured in the presence of RANKL (50 ng/ml) for 5–7 days, followed by TRAP staining (original magnification, × 5). **(B,D**) TRAP-positive multinucleated osteoclasts (nuclei ≥ 3) were counted and the average size of osteoclasts was measured by Image-Pro-Plus 6 software. **(E)** RAW264.7 cells were cultured on Corning Osteo Assay Surface 96-well plates following the aforementioned method to observe the representative images of resorption pits (original magnification, × 5). **(F)** The area and depth of the resorptive hydroxyapatite-coated surface were recorded with stack 3D surface plot in Image-Pro-Plus 6 software. **(G)** The resorption area of the hydroxyapatite-coated surface was analyzed by Image-Pro-Plus 6 software. **(H)** The MMP9 content in OCs induced for 5–7 days in supernatant by ELISA assay. **(I)** Acid secretion was detected by acridine orange staining using a fluorescent microplate reader (Ex = 482 nm and Em = 588 nm). Data are expressed as mean ± SD. *p < 0.05; **p < 0.01; ***p < 0.001. E11 transfections were compared with NC for statistical analysis.

The cell membranes of differentiated and matured OCs are highly polarized. The proton pumps localized on the ruffled border secrete acids, degrade organic bone matrix with releasing hydrolase, and form resorption lacunae on the bone surface (Boyle et al., 2003). siRNA sequence specifically targeting GBF1 and the specific inhibitor GCA thereof in RAW 264.7 cells was used to knock down the expression of GBF1, so as to explore the role of GBF1 on OC differentiation and activation. Compared with negative control, suppression of GBF1 significantly decreased the number and size of TRAP^+^ multinucleated OCs (Figures 1A–D). Additionally, upon stimulation of RANKL, GBF1-mediated bone absorptive activity significantly improved (Figure 1E). Correspondingly, the area and depth of bone erosion were more intuitively represented in the stack 3D surface plot (Figures 1F,G). Subsequently, impaired acid secretion in GBF1 knocking down OCs using acridine orange staining was found, and the extracellular MMP9 content was significantly reduced (Figures 1H,I). The aforementioned data indicate that GBF1 was vital to the activation and functions of OCs *in vitro*.

### Inhibition of GBF1 Reduced RANKL-Mediated Protein Expression

**FIGURE 2:**
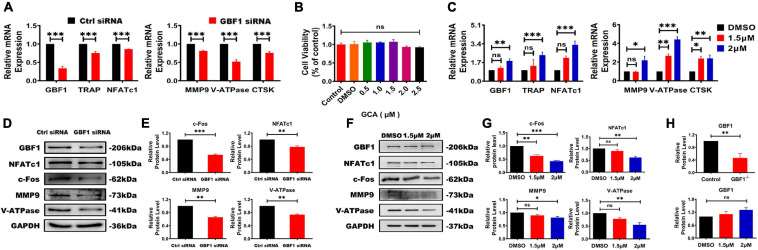
Inhibition of GBF1 reduced the expression of osteoclast-specific gene and protein. **(A,B,H)** The mRNA and protein levels of GBF1, c-Fos, NFATc1, TRAP, NFATc1, MMP9, V-ATPase, and CtsK were analyzed by quantitative real-time PCR and western blot, respectively. The intensity of the blots was quantified using densitometry scan and Image J software. E11 transfection was compared with NC for statistical analysis. **(B)** The effect of GCA (concentration range from 0.5 μM to 2 μM) on RAW264.7 cells viability was measured using the CCK8 assay for 3 days. **(C,F–H)** Cultured RAW264.7 cells in the presence of RANKL (50 ng/ml) were treated with 1.5 and 2 μM GCA for 3 days, and the DMSO group served as the control group. Quantitative real-time PCR and Western blot were used to detect the changes of the aforementioned genes. Data are expressed as mean ± SD of three independent experiments. ^∗^*p* < 0.05; ^∗∗^*p* < 0.01; ^∗∗∗^*p* < 0.001.

To further investigate the effect of GBF1 on osteoclastogenesis, the expression of OC-related transcription factors was subsequently investigated. The present results reveal that transfection with GBF1 siRNA reduced the expression of early OC transcription factor NFATc1 and c-Fos (Figures 2A,D,E). Subsequently, compared with the negative control, the expression of the downstream target genes (TRAP, CtsK, MMP9, and V-ATPase) also decreased (Figures 2A,D,E). Recent studies have provided strong support for GCA as a potent, highly effective, and rapidly reversible small inhibitor likely targeting GBF1 (Pan et al., 2008). In the present study, further verification on whether GCA could impair osteoclastogenesis by inhibiting the expression of OC-specific genes was provided. RAW264.7 cells were first treated with different concentrations of GCA to measure the survival rate. There was no significant difference in cell viability under GCA treatment (Figure 2B). Hence, 1.5, 2, and 2.5 μM were chosen for subsequent experiments. Consistent with previous data, the protein levels of V-ATPase, NFATc1 and MMP9, but not GBF1, were inhibited by 2 μM GCA (Figures 2C,F,G). Unexpectedly, the increase in mRNA levels of these genes was highly variable (Figures 2C,H), indicating that GCA mediated a negative feedback loop, compensatively increased the level of OC activating factors, and maintained cell viability.

### GBF1 Negatively Correlated With COPI Coat Complex and FAM129A

**FIGURE 3:**
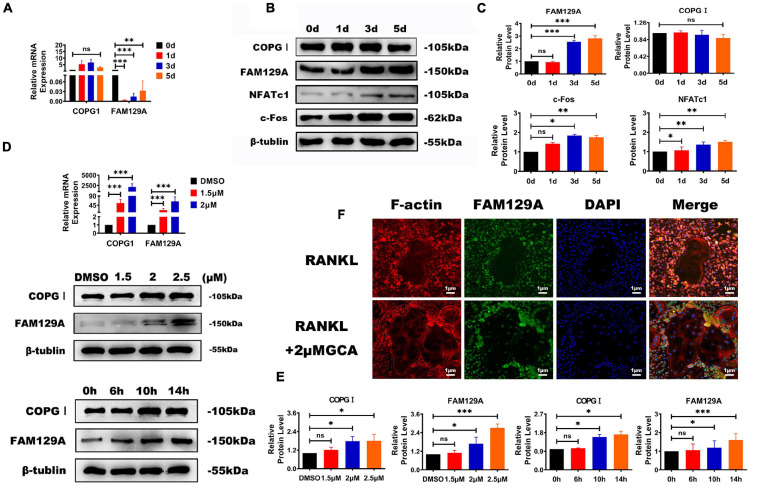
Functional effects of GBF1 on COPGI and FAM129A. **(A–C)** The mRNA expression and protein levels of c-Fos, NFATc1, COPGI, and FAM129A at different stages of osteoclast differentiation were examined by quantitative real-time PCR and Western blot, respectively. The intensity of the blots was quantified using densitometry scan and Image J software. The undifferentiated group (Day 0) was used as the control group. **(D,E)** Cultured RAW264.7 cells in the presence of RANKL (50 ng/ml) were treated with 2 μM of GCA for 0, 6, 10, and 14 h and at 1.5, 2, and 2.5 μM concentration of GCA for 3 days. Quantitative real-time PCR and Western blot were used to analyze the expression of COPGI and FAM129A. **(F)** Osteoclasts derived from RAW264.7 cells were incubated with 2 μM of GCA for 1–2 days, and the actin rings, FAM129A, and nuclei of osteoclasts were stained with phalloidin-FITC, FAM129A antibody, and DAPI, respectively (original magnification, × 20). Data are expressed as mean ± SD of three independent experiments. ^∗^*p* < 0.05; ^∗∗^*p* < 0.01; ^∗∗∗^*p* < 0.001.

The COPI coat complex subunit gamma 1 (COPGI) is a cytoplasmic protein complex that reversibly binds to non-clathrin-coated vesicles budding from the Golgi membrane, and further mediates biosynthetic protein transport between the ER and the Golgi apparatus (Deng et al., 2009). Tumor promotor FAM129A has recently been reported to activate FAK signaling pathway and upregulate MMP2 and MMP9 in tumor tissues (Feng et al., 2019; Zhang et al., 2019). A significant increase in the protein levels of c-Fos, NFATc1, and FAM129A during osteoclastogenesis was detected, while FAM129A GI expression exhibited no difference (Figures 3A–C). To confirm the influential function on COPGI and FAM129A caused by GBF1, different concentrations of GCA were selected to verify the increased protein levels of COPGI and FAM129A in a time-dependent manner, among which 2.5 μM GCA upregulated the expression of FAM129A more strikingly (Figures 3A,D,F). Furthermore, as evidenced by immunofluorescence, higher amounts of endogenous FAM129A were captured in the OC nuclei on the third day within supplementation of 2 μM GCA (Figure 3E). The aforementioned results indicate that GBF1 promoted COPI-mediated vesicle trafficking in OCs and FAM129A might be a downstream effector.

### GBF1-Mediated Suppression of ER Stress and Apoptosis

**FIGURE 4:**
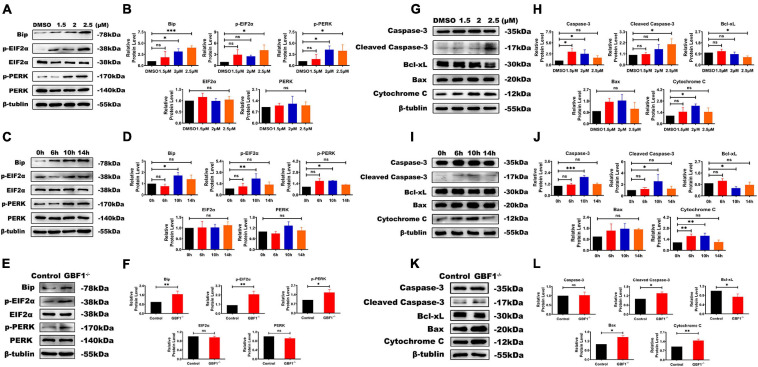
Interference with GBF1 induced osteoclast ERS and autophagy. **(A–D)** Western blot analysis of autophagy-related protein in RANKL-induced osteoclasts after GCA treatment (0, 1.5, 2, and 2.5 μM) and at different time points (0, 6, 10, and 14 h) treated with 2 μM GCA. **(E,F,K,L)** Western blot analysis of autophagy-related protein in RANKL-induced osteoclasts by siRNA transfection. **(E,F)** Western blot analysis of autophagy related proteins in RANKL induced osteoclasts by siRNA transfection.**(G–J)** Western blot analysis of apotosis related proteins induced by GCA. **(K,L)** Western blot analysis of apotosis related proteins after knocking down of GBF1. ^∗^*p* < 0.05; ^∗∗^*p* < 0.01; ^∗∗∗^*p* < 0.001.

In consideration of GBF1 being partly located at ER exit sites, the relationship between GBF1 and ER in OCs was further determined. Western blot analysis data from both knocking down of GBF1 and GCA treatment reveals that the destruction of the GBF1 function upregulated the levels of ERS response-related protein BiP, p-PERK, and p-EIF2α, but not the total expression of PERK and EIF2α (Figures 4A,B,E,F). In addition, the stress level was dosage-dependent, and especially increased after 6 h treatment (Figures 4C,D). Similar to the consequences caused by ERS, the pro-apoptotic protein Bax, Cytochrome C, and Cleaved Caspase-3 were upregulated, while the anti-apoptotic protein Bcl-2 was downregulated (Figures 4G–L). Taken together, the aforementioned results and the time course of the changes suggest that the specific inhibition of GBF1 in OCs had significant effects on the ERS and apoptosis of OCs.

### Effect of GBF1 Depletion on Autophagy and Golgi Apparatus

**FIGURE 5:**
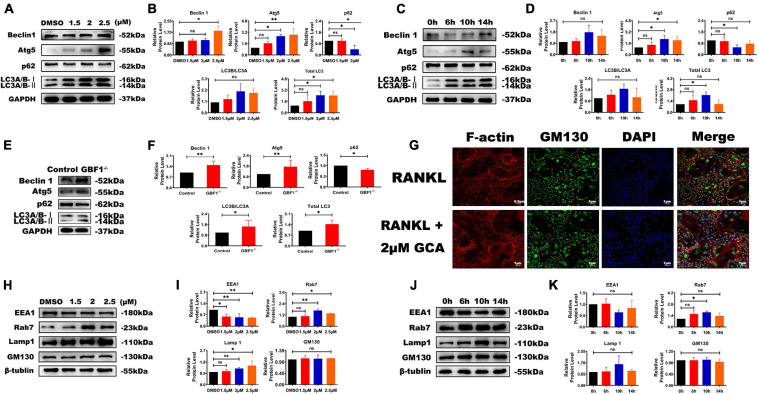
Decrease of GBF1 further caused cell autophagy and affected endosome maturation. **(A–F)** Western blot analysis of autophagy-related protein in RANKL-induced osteoclasts after GCA treatment (0, 1.5, 2, and 2.5 μM) and at different time points (0, 6, 10, and 14 h) treated with 2 μM GCA or with siRNA. **(G)** Immunostaining of actin rings, GM130 antibody, and nuclei in osteoclasts treated with 2 μM of GCA for 1–2 days (original magnification, × 20). The red arrows represent the perinuclear Golgi apparatus. **(H–K)** Western blot analysis of endosome-related protein in RANKL-induced osteoclasts after GCA treatment (0, 1.5, 2, and 2.5 μM) and at different time points (0, 6, 10, and 14 h) treated with 2 μM GCA. Data are expressed as mean ± SD of three independent experiments. ^∗^*p* < 0.05; ^∗∗^*p* < 0.01; ^∗∗∗^*p* < 0.001.

A complex interaction exists between autophagy and apoptosis (Song et al., 2017). In previous research by the present authors, the effect of GBF1 on ERS and apoptosis was confirmed, and further hypothesis was made in terms of whether GBF1 regulated the autophagy process of OCs. As expected, suppression of GBF1 in OCs increased the protein levels of Beclin1, Atg5, and LC3, and the expression of p62 was reduced in a dosage-dependent manner *in vitro* (Figures 5A,B,E,F), in line with the increased autophagy activity at 6 h (Figures 5C,D). Subsequently, the levels of several proteins involved in endosomes were measured, such as early endosomal marker (EEA1), secondary endosomal marker (Rab7), and late endosomal marker (LAMP1). The expression levels of Rab7 and LAMP1 were significantly increased in OCs, which was contrary to the level of EEA1 (Figures 5H–K). Notably, GM130 staining was found to be widely scattered and the Golgi apparatus was extensively dispersed in GBF1-deficient OCs (Figure 5G). The aforementioned results further validated that GBF1 deficiency enhanced autophagy activity, possibly by affecting endosome maturation and the Golgi apparatus.

## Discussion

Osteoclasts are central in all three fundamental processes of bone biology, namely, endochondral ossification during development, bone modeling during growth, and the subsequent remodeling in adulthood (Jacome-Galarza et al., 2019). However, over-activation of OCs leads to enhanced bone resorption and osteopenia, along with the risk of numerous osteolytic diseases (Sun et al., 2019). The RANKL/RANK pathway is integral for both OC formation and function, which are thought to be mediated by the common transcription factor c-Fos and NFATc1 (Takayanagi, 2007). In the present study, the results from genetic approaches and drug inhibition were combined, and GBF1 deficiency was found to contribute to the degradation of OC-associated proteins, including c-Fos, NFATc1, TRAP, V-ATPase, MMP9, and CtsK, further confirming the suppression of GBF1 on OC activity and bone resorption *in vitro*. Unexpectedly, there was no change in the protein level of GBF1 with GCA treatment for 14 h, while the mRNA levels of OC-related factors significantly increased, indicating that GCA had no effect on the translation level of GBF1 or the effects thereof gradually disappeared. The effect of GCA was rapidly reversible, causing Golgi reassembly and protein secretion restoration within 15 min and 1 h of removing the compound, respectively. Thus, the most reasonable explanation is that the rapid inhibitory effect of GCA on GBF1 had a long-term effect on the downstream signaling pathways (Supplementary Figures 1A–C).

Vesicular trafficking is the basis of the lifespan of OCs, governing cell communication *via* secretion and the uptake of signaling molecules, enzymes, and adhesion molecules, The large size and abundant organelles of OCs are strong evidence for that (Coxon and Taylor, 2008). COPI proteins mediate a retrograde transport pathway that selectively recycles proteins from the *cis*-Golgi complex to the ER. Additionally, COPI coat proteins have complex functions in intra-Golgi trafficking and maintaining the normal structure of the mammalian interphase Golgi complex. The present finding also reveals that GBF1 suppression triggered rapid dispersion of COPI from Golgi membranes, in line with the increased protein level of COPI after the addition of GCA. Furthermore, inhibition of GBF1 resulted in OC swelling in the middle differentiation stage, with significantly increased vacuoles and volume, whereas the actin ring did not respond to GBF1 deficiency. The aforementioned findings are presumably regulated by GBF1-mediated vesicle trafficking, which can target the ARFs family for activation. Contrarily, through replacing GDP with GTP and membrane positioning mediated by COPI, GBF1 inactivation impaired the conversion of activated ARFs. The changes likely resulted in the blocked transport and accumulation of secretory proteins in OCs. Notably, GBF1 is not an OC-specific marker, so the function thereof in osteoclastogenesis might be related to conventional vesicular trafficking, but is not a physiological mechanism of OC.

GBF1 functions in regulating the secretion of soluble proteins and anchored membrane proteins between endoplasmic reticulum and Golgi apparatus in an orderly manner; thus, the functional inactivation thereof will aggravate ERS (Dorobantu et al., 2014). As such, the contents of main ERS proteins after treatment with GCA were further examined, and western blot results reveal that the expression levels of BiP, p-PERK, and p-EIF2α were increased. Significantly, FAM129A, as a tumor biomarker, was found to be critical in mediating the main transcriptional effectors of UPR. FAM129A was elevated in both the presence of GBF1 inhibitor and the differentiation process of OCs, but the two seemed to be contradictory. After GBF1 knockdown, the expression of FAM129A was upregulated, but the MMP9 protein level was decreased, as well as the secretion thereof to the extracellular, which was different from that in tumor cells. Additionally, through positive and negative feedback loops, the PERK–EIF2α pathway was regulated, allowing cells to fine-tune the response thereof to incoming pressure according to the environment.

Endoplasmic reticulum stress stimulates the production of autophagy to reduce the degree of swelling, relieving the pressure of protein accumulation in the ER and finally restoring the normal state of ER and keeping the cells alive (Senft and Ronai, 2015). In addition, the Golgi serves as a membrane source during autophagosome development, GCA-induced dissociation, and dispersal of the Golgi and TGN. *Via* trafficking independent mechanisms that involve inactivation of mTOR signaling, the impaired ARF-dependent assembly of coated vesicles can stimulate autophagy (Sáenz et al., 2009). The present data demonstrates that the ERS–autophagy axis had a vital function in the differentiation of GBF1-inhibited OCs. Immunofluorescence staining shows GCA-induced dissociation and dispersion of the Golgi apparatus. Further analysis results indicate that GCA promoted autophagy of OCs by regulating the expression of p-mTOR, Beclin1, and Atg5. An increase of both LC3 and LAMP1 was also observed, which are biomarkers in monitoring autophagy and the degradation of autophagy substrate p62. In summary, the *in vitro* results reveal that inhibition of GBF1 destroyed ER and the Golgi structure, inhibited assembly of budding vesicles and the transportation of secreted proteins, and then initiated autophagy.

By the process of autophagy, lysosomes are responsible for the degradation of intracellular components. Newly formed autophagosomes undergo a stepwise maturation by fusing with endosomes and lysosomes. Such process was manifested in the presence of Rab7-positive late endosomes causing the formation of aggregates and tubular structures, while the effects of Rab5-positive early endosomes appeared to be less prominent (Ackema et al., 2013). Similarly, the present results reveal increased expression in Rab7 and decreased expression in EEA1, indicating that more early endosomes are transformed into late endosomes, and thus autolysosome formed when GBF1-regulated autophagy occurred. In the late stage of differentiation, due to sustained or strong ERS and excessive autophagy, autophagy apoptosis of OCs increased, which was consistent with the decrease of bone resorption.

In conclusion, GBF1 was demonstrated to be a pivotal regulator of both ERS and autophagy in OC proliferation and differentiation through synergistic stimulation of the expression of p-mTOR, Atg5, and LC3, as well as p-PERK, p-EIF2α, and FAM129 regulation. The aforementioned results implicate that GBF1 is a putative therapeutic target for pathological bone loss.

## Data Availability Statement

The original contributions presented in the study are included in the article/Supplementary Material, further inquiries can be directed to the corresponding author/s.

## Author Contributions

CW and YZ designed this study, carried out most of the experiments, and wrote the manuscript. YX and HT helped to do some of the cell culture work. CP, HQL, KL, LW, HL, and TQ helped to collect samples and some western blotting. CH helped to confocal microscope imaging. CL contributed to the revision of this manuscript. CZ supervised the experiments, analyzed the results, and proofread the manuscript. All authors contributed to the article and approved the submitted version.

## Conflict of Interest

The authors declare that the research was conducted in the absence of any commercial or financial relationships that could be construed as a potential conflict of interest.

## Publisher’s Note

All claims expressed in this article are solely those of the authors and do not necessarily represent those of their affiliated organizations, or those of the publisher, the editors and the reviewers. Any product that may be evaluated in this article, or claim that may be made by its manufacturer, is not guaranteed or endorsed by the publisher.

## Supplementary Material

Supplementary Figure 1 The influence of GCA on GBF1 expression. **(A)** Relative mRNA expression of GBF1 was examined at different stages of osteoclasts differentiation. The undifferentiated group (day 0) was used as a control group. **(B)** Western blot analysis of GBF1 in osteoclasts treated with 2 μM GCA at different time points (0, 6, 10, and 14 h). **(C)** Immunostaining of actin rings, GBF1 antibody and nuclei in osteoclasts treated with 2 μM of GCA for 1–2 days (original magnification, × 20). Data are expressed as mean ± SD of three independent experiments. ^∗^, *p* < 0.05; ^∗∗^, *p* < 0.01; ^∗∗∗^, *p* < 0.001.

Supplementary Table 1 PCR primer list for quantitative real-time PCR.
