# Supplementary figures and images for: The Regulatory Role of GBF1 on Osteoclast Activation Through EIF2a Mediated ER Stress and Novel Marker FAM129A Induction
Source: Front Cell Dev Biol. 2021 Aug 25;9:706768. doi: 10.3389/fcell.2021.706768 (PMC8424197; doi:10.3389/fcell.2021.706768)

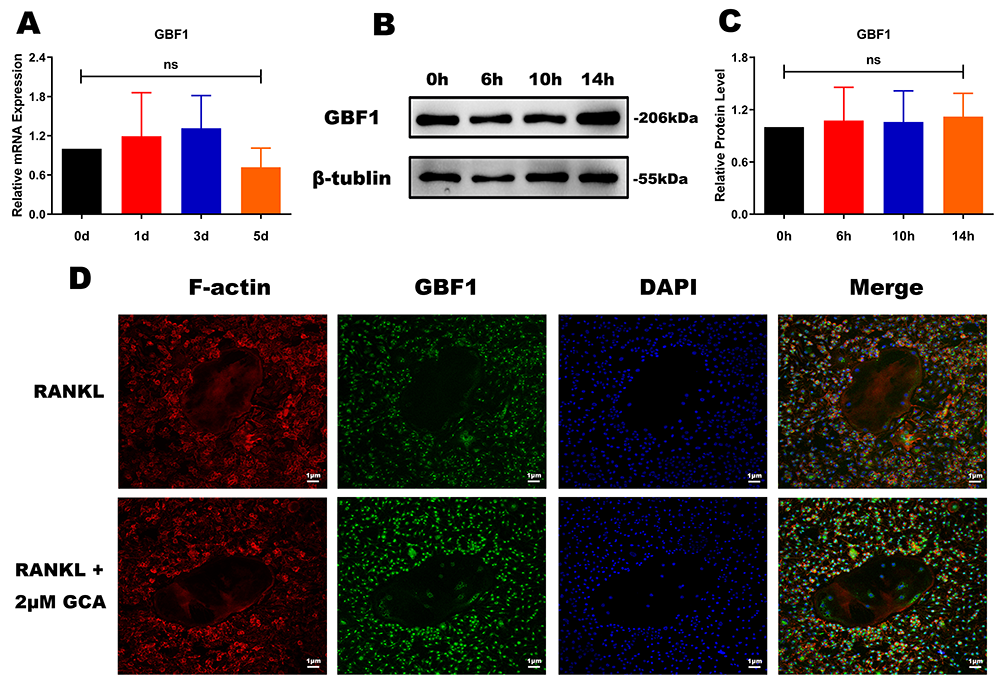

Supplement: Supplementary Figure 1 — The influence of GCA on GBF1 expression. (A) Relative mRNA expression of GBF1 was examined at different stages of osteoclasts differentiation. The undifferentiated group (day 0) was used as a control group. (B) Western blot analysis of GBF1 in osteoclasts treated with 2 μM GCA at different time points (0, 6, 10, and 14 h). (C) Immunostaining of actin rings, GBF1 antibody and nuclei in osteoclasts treated with 2 μM of GCA for 1–2 days (original magnification, × 20). Data are expressed as mean ± SD of three independent experiments. ∗, p < 0.05; ∗∗, p < 0.01; ∗∗∗, p < 0.001. [file Image_1.TIF]
