# Supplementary material for: The Regulatory Role of GBF1 on Osteoclast Activation Through EIF2a Mediated ER Stress and Novel Marker FAM129A Induction
Source: Front Cell Dev Biol. 2021 Aug 25;9:706768. doi: 10.3389/fcell.2021.706768 (PMC8424197; doi:10.3389/fcell.2021.706768)
Supplement: Supplementary Table 1 — PCR primer list for quantitative real-time PCR. [file Table_1.pdf]

All the primers used in this study.

| Gene name | Forward primer sequerence (5'-3') | Reversed primer sequerence (5'-3') |
|---|---|---|
| GBF1 | GAGATCACCACCAGTGAGCC | CCTTCCTGCTGAGGGTCAAG |
| TRAP | TCTCCAAGCGATGGAACTACC | TTCCGCACAACTCGACTGTA |
| NFATc1 | AGGACCCGGAGTTCGACTT | CATAACTGTAGTGTTCTGCGGC |
| MMP9 | AAGGCAGCGTTAGCCAGAAG | GCGGTACAAGTATGCCTCTGC |
| CtsK | TCCGAAAAGAGCCTAGCGAA | CCGAGAGATTTCATCCACCTTG |
| V-ATPase | CTGGTTCGAGGATGCAAAGC | GACCAAGCTCCTACGTTTCG |
| COPG? | AGCAATTGTGGACAAGGTTCC | ATGATGTTGTCACTGGACGC |
| FAM129A | GCGACGCCTCTCAAGGAAGTT | AGGCAGAGGACTGACACCAAC |
| β-actin | ACCGTGAAAAGATGACCCAG | GTACGACCAGAGGCATACAG |
